# Health Tract, No. 296

**Published:** 1867-08

**Authors:** 


HEALTH TRACT, No. 296.

### HEMORRHAGE,

Is literally "blood flowing," from any part of the body, but it is commonly used in a restricted sense to mean spitting blood from the lungs, which is usually regarded, and is, the death knell of the individual, sooner or later, from consumption of the lungs; hence, when any person "spits blood" he tries to persuade himself that it is from the throat or from the gums or from the nose. In very many cases it is a harmless occurrence in women, and sometimes in men; but as to the latter, it almost always means death from consumption. If the quantity discharged does not exceed a few table-spoonfuls, it is best to let it alone; to remain quiet and composed on the bed, and to eat bits of ice with avidity; this cools the stomach and draws the blood there, away from the lungs; besides, it lowers the heat of the body, tends to cool the blood, lessens its fluidity and makes it flow more slowly. A very common remedy is to eat common salt, which is perhaps the very best thing that can be done, in addition to ice, and be quiet, because, first, it is always at hand, is perfectly safe, and by its thirst-exciting and nauseating tendency diverts the blood from the lungs and diminishes its fluidity; cloths dipped in ice water and laid on the chest, and replaced by others every minute or two, is very efficient, especially if the feet are kept half-leg deep in water as hot as can be borne; any, and all these things do good in another direction, they help to quiet the mind, to compose the patient, and soothe the pertubation which is usually excited by such an occurrence, While these things are doing, send for a physician and turn the whole case over to him, and allow him to assume all its responsibilities.

When blood is loose in the lungs, or its vessels are gorged with it, it is better out than in, and it keeps off cough or moderates it very greatly; hence it is temporarily curative, and does an immediate and unmixed good if it does not exceed a tea-spoonful every few days. It should be borne in mind, that in many cases it arises from the want of a vigorous general circulation of the blood, and whatever promotes that tends to arrest the hemorrhage. John Randolph was afflicted thus for many years and as soon as he was attacked, he would jump on his mule or horse and trot off for an hour or two. N. P. Willis did the same thing with admirable effect.

